# Liver Fibrosis Index-4 Does Not Correlate to Liver Elastography in Patients with Inflammatory Bowel Disease

**DOI:** 10.3390/jcm13216430

**Published:** 2024-10-27

**Authors:** Iván Ferraz-Amaro, Alejandro Hernández-Camba, Marta Carrillo-Palau, Noemi Hernández Álvarez-Buylla, Antonia de Vera-González, Alejandra González-Delgado, Elena Heras-Recuero, Miguel Á. González-Gay

**Affiliations:** 1 Division of Rheumatology, Hospital Universitario de Canarias, 38320 Tenerife, Spain; 2 Department of Internal Medicine, Universidad de La Laguna (ULL), 38200 Tenerife, Spain; 3 Division of Gastroenterology, Hospital Universitario de Nuestra Señora de la Candelaria, 38010 Tenerife, Spain; ahercamxl@gobiernodecanarias.org; 4 Division of Gastroenterology, Hospital Universitario de Canarias, 38320 Tenerife, Spain; mcarpal@gobiernodecanarias.org (M.C.-P.);; 5 Division of Central Laboratory, Hospital Universitario de Canarias, 38200 Tenerife, Spain; avergonq@gobiernodecanarias.org (A.d.V.-G.); agondele@gobiernodecanarias.org (A.G.-D.); 6 Division of Rheumatology, IIS-Fundación Jiménez Díaz, 28040 Madrid, Spain; elena.herasr@quironsalud.es; 7 Department of Medicine, University of Cantabria, 39011 Santander, Spain

**Keywords:** inflammatory bowel disease, ulcerative colitis, Crohn’s disease, metabolic dysfunction-associated steatotic liver disease, fibrosis-4 (FIB-4) index, elastography

## Abstract

**Background**: Inflammatory bowel disease (IBD) is associated with an increased prevalence of metabolic dysfunction-associated steatotic liver disease (MASLD). The Fibrosis-4 (FIB-4) index is a non-invasive tool for assessing liver fibrosis that has been validated in various liver diseases. The main objective of this study was to study whether the FIB-4 index is a reliable predictor of liver fibrosis, as assessed through elastography, in patients with IBD. We additionally aimed to analyze if FIB-4 associates with IBD characteristics such as lipid profile, subclinical carotid atherosclerosis, and insulin resistance indices. **Methods**: A cross-sectional study was conducted, enrolling 197 patients with IBD. Subjects underwent comprehensive clinical and laboratory evaluations. Hepatic fibrosis was assessed non-invasively using the FIB-4 index and transient elastography, while abdominal ultrasonography was performed to grade hepatic steatosis based on the degree of fat infiltration. To investigate the associations between disease characteristics and FIB-4 score and the correlation of this index to elastography, a multivariable linear regression analysis was conducted. **Results**: The presence of diabetes, hypertension, and metabolic syndrome was associated with significantly higher FIB-4 levels. However, FIB-4 did not show a relationship with disease characteristics such as phenotype or activity indices. Furthermore, FIB-4 did not demonstrate a correlation with liver stiffness values measured by elastography. **Conclusions**: Our findings suggest that the FIB-4 index may not be a reliable tool for assessing hepatic fibrosis in patients with IBD. This observation is particularly significant given the high prevalence of MASLD in the IBD population.

## 1. Introduction

Inflammatory bowel disease (IBD) consists of two main conditions: ulcerative colitis (UC) and Crohn’s disease (CD). While UC is confined to the colon, CD can affect any part of the gastrointestinal tract. Both disorders involve chronic inflammation and follow a relapsing–remitting pattern. In UC, inflammation is limited to the colon’s mucosal layer, while CD is depicted by transmural inflammation and the presence of skip lesions. The pathogenesis of IBD is thought to be multifactorial [1]. Extraintestinal manifestations of IBD can appear at diagnosis or develop as the disease progresses, affecting multiple organs and systems such as the skin, joints, hepatobiliary tract, eyes, and kidneys [2].

Hepatobiliary manifestations are usual in patients with IBD, with approximately 30% experiencing abnormal liver function tests and around 5% developing chronic liver diseases [3]. The mechanisms linking liver complications to IBD may involve shared autoimmune pathways (as seen in IgG4-related cholangitis, primary sclerosing cholangitis, and autoimmune hepatitis), intestinal inflammation (such as granulomatous hepatitis and portal vein thrombosis), metabolic dysfunction (including non-alcoholic fatty liver disease or cholelithiasis), or adverse effects of medications (such as drug-induced liver injury or hepatitis B virus reactivation) [3].

Metabolic dysfunction-associated steatotic liver disease (MASLD), previously identified as nonalcoholic fatty liver disease, is one of the most common liver disorders worldwide, affecting approximately 25% of the world’s population [4]. MASLD is a predisposing factor for the progression of liver fibrosis and the eventual development of cirrhosis [5]. This seems also to be the case in patients with IBD, who appear to have a greater predisposition to MASLD, with prevalence rates reaching almost 40% [6,7,8,9]. The main risk factor for MASLD in the general population is metabolic syndrome. However, IBD patients are at risk of developing MASLD even with fewer metabolic risk factors. IBD-specific factors that contribute to this increased risk include history of small bowel surgery, disease severity and duration, dependence on parenteral nutrition, and use of high-dose corticosteroids [10].

The Fibrosis-4 (FIB-4) index, a non-invasive scoring system used to estimate liver fibrosis, was first introduced in 2006 [11,12]. The FIB-4 index is calculated using platelet count, alanine aminotransferase (ALT), aspartate aminotransferase (AST), and age. Originally developed to assess liver fibrosis in patients with viral hepatitis, it has also proven useful in MASLD. For example, a study comparing non-invasive fibrosis markers in patients with MASLD found that the FIB-4 index was superior to seven other non-invasive markers in this setting [13]. The FIB-4 index has demonstrated validity in assessing fibrosis in MASLD, as supported by practice guidelines and meta-analyses on this topic [14,15,16].

To date, the performance of the FIB-4 index in patients with IBD has not been extensively investigated. In this study, we explored the relationship between FIB-4 score and a wide range of disease characteristics, including IBD manifestations, cardiovascular disease factors, insulin resistance indices, and lipid profiles. Additionally, we examined the correlation between the FIB-4 score and the presence of liver fibrosis, measured by elastography, in patients with IBD. This comprehensive approach aims to improve our understanding of the utility and implications of FIB-4 within the IBD population.

## 2. Materials and Methods

### 2.1. Study Participants

This cross-sectional work was performed with 197 consecutive IBD adult patients (>18 years). All participants were under the care of gastroenterology specialists and received regular follow-up evaluations at outpatient digestive health clinics. To participate in the study, an IBD diagnosis based on clinical, endoscopic, and histological assessments was necessary. Additionally, patients needed to have a minimum duration of the disease higher than one year. Patients were excluded from the study if they had a history of malignancy or any other chronic inflammatory or autoimmune disorders or signs of an active infection. Furthermore, patients with autoimmune hepatitis, primary sclerosing cholangitis, alcoholism, or viral hepatitis infection were omitted. The research procedure was reviewed and approved by the Ethics Committees of both Hospital Universitario de Canarias and Hospital Universitario Nuestra Señora de La Candelaria (approval reference: CHUC_2019_103). Prior to their participation, all subjects provided written informed consent. This study involving human participants was conducted in full compliance with the ethical principles outlined in the Declaration of Helsinki.

### 2.2. Data Collection

Surveys were administered to IBD patients to collect details regarding their clinical history, cardiovascular risk factors, and medication use. Hypertension was characterized by a systolic blood pressure greater than 140 mmHg or a diastolic pressure exceeding 90 mmHg. For those with CD, disease activity was assessed using the Crohn’s Disease Activity Index (CDAI) and the Harvey–Bradshaw Index [17]. The CDAI was divided as follows: 0 to 149 points for asymptomatic remission, 150 to 220 points for mildly to moderately active disease, 221 to 450 points for moderately to severely active disease, and 451 to 1100 points for severely active to fulminant disease. The Harvey–Bradshaw Index was classified into 0 to 4 points for clinical remission, 5 to 7 points for mildly active disease, 8 to 16 points for moderately active disease, and 17 to 100 points for severely active disease. UC activity was evaluated using the partial Mayo Clinic score [18]. Data were gathered on the treatments used for the disease, including mesalazine, prednisone (recorded either as a binary variable or in mg/day), azathioprine, methotrexate, and biologic therapies.

The presence of metabolic syndrome was determined using the National Cholesterol Education Program (NCEP/ATPIII) criteria [19]. Metabolic syndrome, according to the NCEP ATP III criteria, is diagnosed if three or more of the following five conditions are present: waist circumference greater than 102 cm for men or 88 cm for women, blood pressure exceeding 130/85 mmHg, fasting triglyceride levels above 150 mg/dL, fasting high-density lipoprotein (HDL) cholesterol levels below 40 mg/dL for men or 50 mg/dL for women, and fasting blood glucose levels over 100 mg/dL.

The Systematic Coronary Risk Evaluation 2 (SCORE2) tool for cardiovascular risk was estimated following established methods, factoring in variables such as gender, age, smoke status, systolic blood pressure, and non-HDL cholesterol values [20]. SCORE2 predicts a subject’s 10-year risk of both fatal and non-fatal cardiovascular events in adults between the ages of 40 and 69. For individuals aged 70 and older, the SCORE2-OP (Older Persons) model is used to estimate 5-year and 10-year risks for both fatal and non-fatal cardiovascular disease events.

### 2.3. Laboratory Assessments

The erythrocyte sedimentation rate (ESR) was assessed through the Westergren method, while C-reactive protein (CRP) levels were quantified through a high-sensitivity immunoassay. Cholesterol, HDL cholesterol, and triglyceride levels were measured via an enzymatic colorimetric technique (Roche, Barcelona, Spain). Apolipoproteins and lipoprotein (a) were analyzed using a quantitative immunoturbidimetric method (Roche, Barcelona, Spain). Cholesterol levels varied between 0.08 and 20.7 mmol/L, with an intra-assay coefficient of variation of 0.3%. Triglycerides oscillated from 4 to 1000 mg/dL, with an intra-assay variation coefficient of 1.8%, and HDL cholesterol ranged from 3 to 120 mg/dL, with an intra-assay variation coefficient of 0.9%. The atherogenic index was calculated using the total cholesterol to HDL ratio based on the Castelli formula, while LDL cholesterol was estimated using the Friedewald formula. Dyslipidemia was characterized by the presence of one or more of the following conditions: total cholesterol exceeding 200 mg/dL, triglycerides over 150 mg/dL, HDL cholesterol under 40 mg/dL in males or below 50 mg/dL in females, or LDL cholesterol levels superior to 130 mg/dL.

The homeostatic model assessment (HOMA) method was utilized to evaluate insulin resistance (IR). In brief, the HOMA model estimates insulin sensitivity (%S) and β-cell function (%B) using fasting plasma insulin, C-peptide, and glucose levels. This study employed HOMA2, the updated computer version of the HOMA mode [21]. HOMA2 allows for the assessment of insulin sensitivity and beta-cell function using paired fasting plasma glucose and either specific insulin or C-peptide concentrations. The model accommodates insulin levels ranging from 1 to 2200 pmol/L and glucose levels from 1 to 25 mmol/L. C-peptide is considered a more reliable indicator of β-cell function due to its secretion pattern, while insulin data is preferred for %S calculations, as HOMA2-%S is based on glucose clearance relative to insulin concentration. In our work, serum insulin levels were used to determine IR and %S, whereas serum C-peptide levels were utilized for %B calculations. The computerized model provided an insulin sensitivity value, stated as HOMA2-%S (with 100% representing normal insulin sensitivity). HOMA2-IR, the index of insulin resistance, was calculated as the inverse of %S.

### 2.4. Liver Disease Assessments

FIB-4 was estimated through the equation: FIB-4 = Age × AST / (0.001 × Platelets × square root (ALT)), where ALT is alanine aminotransferase and AST is aspartate aminotransferase [12]. The cut-off values established for fibrosis risk are as follows: low risk is defined as less than 1.45 points, indeterminate risk ranges from 1.45 to 3.25 points, and high risk is greater than 3.25 points. Hepatic fibrosis was evaluated non-invasively using transient elastography, also known as Fibroscan^®^ (version 2024, Echosens, Paris, France). The assessment protocol required a minimum of ten valid readings, with result validation contingent upon achieving a success rate of at least 60% and maintaining an interquartile range below 30%. Based on these measurements, liver fibrosis was classified on a scale ranging from F0, indicating no fibrosis, to F4, signifying cirrhosis. The correlation between values of Fibroscan^®^ and liver fibrosis stages is as follows: less than 7.6 KPa corresponds to F0-F1, 7.7–9.4 KPa corresponds to F2, 9.5–14 KPa corresponds to F3, and greater than 14 KPa corresponds to F4 [22,23]. Both abdominal ultrasound and elastography procedures were performed after 6 h of fasting.

Abdominal ultrasonography was conducted on patients with IBD using B mode to assess the extent of steatosis based on the level of fat infiltration. The degree of fat infiltration was categorized into three levels as previously described [24,25]: mild, characterized by a slight diffuse increase in hepatic echogenicity, with clear visibility of the diaphragmatic line and intrahepatic vascular structures; moderate, where hepatic echogenicity appeared intermediate compared to that of the kidney, accompanied by mild attenuation of the diaphragmatic wall and intrahepatic vessels; and severe, marked by a substantial difference in echogenicity between the liver and kidney, absence of diaphragm visualization, and attenuation of vessels, rendering them unobservable at the hepatic posterior pole.

### 2.5. Carotid Ultrasound Evaluation

We performed an ultrasound examination of the carotid arteries to evaluate two key aspects: the thickness of the intima–media layer in the common carotid artery (cIMT) and the presence of any localized atherosclerotic plaques within the extracranial carotid vasculature. The examination utilized an Esaote MyLab 70 ultrasound system (Genoa, Italy) equipped with a high-frequency linear transducer (7–12 MHz). Measurements were obtained using the software Quality Intima Media Thickness (QIMT) version 6 (Esaote, Genoa, Italy), an automated radiofrequency-based technique settled by Esaote in Maastricht, Netherlands. Our assessment adhered to the Mannheim consensus criteria for plaque identification, focusing on the accessible segments of the extracranial carotid system. This comprehensive examination included the common carotid artery, the carotid bulb region, and the internal carotid artery. According to these guidelines, a plaque was characterized as a focal protrusion into the arterial lumen meeting specific criteria: a cIMT measurement greater than 1.5 mm, a localized thickening at least 50% larger than the surrounding cIMT, or an encroachment on the arterial lumen exceeding 0.5 mm [26].

### 2.6. Statistical Analysis

We summarized the study population’s demographic and clinical profile using different statistical approaches. For categorical data, we reported frequencies. Continuous variables were presented as either mean values with their corresponding standard deviations (SD) or, in cases where the data did not follow a normal distribution, as median values accompanied by their interquartile ranges (IQR). Correlation analysis was performed using the Pearson correlation coefficient. The association between FIB-4 and disease-related factors was examined using linear multivariable regression analysis. Confounding variables were chosen from demographic characteristics and established cardiovascular risk factors based on their univariate associations with FIB-4 (*p* < 0.20). Statistical analyses were performed using Stata software version 17/SE (StataCorp, College Station, TX, USA). All tests were two-sided, with a significance level set at 5%. Results were considered statistically significant when *p* < 0.05.

## 3. Results

### 3.1. Demographics and Disease-Related Data

This study included 197 individuals with an average age of 49 ± (SD) 10 years. The mean FIB-4 score was 0.93 ± 0.42. After categorization, 89% of patients were labeled as having a low risk of fibrosis, while 11% fell into the indeterminate risk category according to this index. The study population had an average BMI of 27 ± 5, with obesity (defined as BMI ≥ 30) affecting 28% of the subjects. An examination of cardiovascular risk factors revealed that 20% were current tobacco users, 6% had diabetes, and 18% had hypertension. Furthermore, 4% of participants were taking aspirin, while 11% were on statins (Table 1).

The cohort comprised 66% CD patients and 34% UC patients. The median disease duration was 12 years (IQR 8–19). In CD patients, ileal involvement and non-stricturing, non-penetrating phenotypes predominated. Disease activity assessment yielded a median CDAI score of 39 (IQR 7–80), with 89% of patients in asymptomatic remission. The HBI corroborated these findings, with a median score of 2 (IQR 0–4) and 82% of patients meeting remission criteria. Among UC patients, 52% had pancolitis. Disease activity evaluation revealed that 78% had a partial Mayo score of less than 2 points, indicative of mild disease or remission. Additional comprehensive disease-specific data are presented in Table 1.

### 3.2. Demographics and Inflammatory Bowel Disease Data Relation to the FIB-4 Index

The relationship between demographics, disease characteristics, and FIB-4 values is shown in Table 2. Age was associated with significantly higher FIB-4 index levels, which is expected given that age is a component of the FIB-4 formula. Conversely, while body mass index (BMI) and abdominal and hip circumferences did not show a direct association with FIB-4, the ratio of these measurements was significantly related to higher FIB-4 values. Regarding cardiovascular risk factors, the presence of diabetes, hypertension, metabolic syndrome, and the use of statins were associated with significantly higher FIB-4 levels. In contrast, smoking, dyslipidemia, obesity, and aspirin use did not demonstrate a significant relationship with the FIB-4 index (Table 2).

The relationship between disease characteristics and FIB-4 values became negligible after adjusting for covariates such as age, sex, diabetes, hypertension, statin use, and waist-to-hip ratio. Specifically, the type of disease, CRP levels, disease phenotypes, activity indices, fecal calprotectin levels, and various treatments were not significantly associated with FIB-4 values (Table 2).

### 3.3. Relationship Between Cardiovascular Disease Data and FIB-4 Values

The relation of cardiovascular-related data to FIB-4 is shown in Table 3. A significant correlation was observed between FIB-4 levels and the SCORE2 calculator results, both when analyzed as continuous variables and when categorized into groups. Since SCORE2 incorporates several variables that are also included in the multivariable adjustment, no additional correction for covariates was performed in this analysis.

The presence of carotid plaque and cIMT were significantly associated with the FIB-4 index in univariable analysis. However, this relationship was lost after adjusting for other cardiovascular risk factors (Table 3). The association between glucose levels, insulin, C-peptide, insulin resistance indices, and beta-cell function indices with FIB-4 was analyzed only for non-diabetic subjects with fasting glucose levels below 110 mg/dL. In this regard, even with univariable analysis alone, no association was demonstrated. This was also the case for the lipid profile. No associations were found between lipid-related molecules and FIB-4 after multivariable adjustment (Table 3).

### 3.4. FIB-4, Liver Stiffness, and Steatosis in IBD Patients

Table 4 shows data on FIB-4 scores, liver stiffness measured by Fibroscan^®^, and steatosis grades assessed via ultrasound in subjects with IBD. Of note, none of the patients had a FIB-4 score indicating high-risk fibrosis. Liver stiffness, as assessed by elastography, had a mean value of 5.27 ± 2.89 kPa. Upon categorization, 86% of patients showed no or mild fibrosis (F0-F1), while 9%, 3%, and 2% had significant fibrosis, severe fibrosis, and cirrhosis, respectively. The overlap between low-risk FIB-4 and elastography F0-F1 was 91%. Ultrasound findings indicated that 51% of IBD patients had no hepatic steatosis, while 31% had mild, 16% had moderate, and 2% had severe steatosis (Table 4).

**Figure 1:**
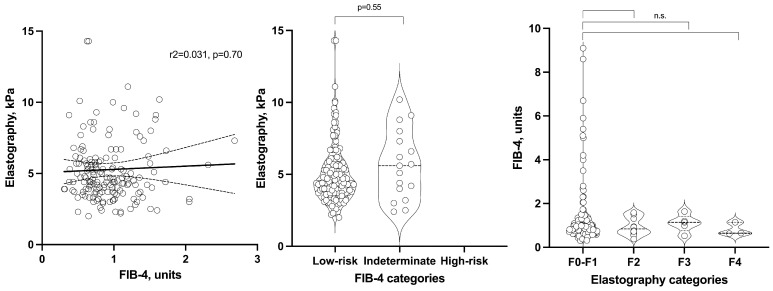
The relationship between index FIB-4 and liver stiffness through elastography is considered both continuous and categorical. Solid and dashed lines represent linear regression line and 95% confidence interval. Non-significant is depicted as n.s.

The relationship between the FIB-4 index and liver elastography measurements was analyzed bidirectionally, with each variable considered as both an independent and dependent variable (Table 4 and Figure 1). When both variables were treated as continuous, no significant associations were observed between FIB-4 scores and elastography values. Similarly, FIB-4 categories did not demonstrate a significant relationship with elastography measurements when the latter was analyzed as a continuous variable. This lack of association persisted when elastography results were categorized. Notably, FIB-4 values in the significant fibrosis, severe fibrosis, and cirrhosis categories did not differ significantly from those in the reference category of no or mild fibrosis (Table 4 and Figure 1).

Regarding ultrasound-based grading of hepatic steatosis, patients classified as having mild, moderate, or severe steatosis did not exhibit significantly different FIB-4 values compared to subjects without steatosis. With respect to elastography measurements, patients with moderate steatosis demonstrated higher liver stiffness values. However, this increase in stiffness was not observed in patients with severe steatosis when compared to those without steatosis (Table 4 and Figure 1).

According to this data, among 127 patients with IBD who had a low-risk FIB-4 score, 18 exhibited elastography values of F2 or higher. This indicates that for every seven normal FIB-4 values, one patient would have a pathological Fibroscan result.

## 4. Discussion

Our study is the first to investigate the performance of the FIB-4 index in a large, well-characterized cohort of patients with IBD. According to our findings, the FIB-4 index is associated with cardiovascular risk factors such as diabetes, hypertension, and metabolic syndrome in this patient population. However, FIB-4 showed no relationship with disease characteristics, including phenotype or activity indices. Additionally, no significant association was found between FIB-4 and liver stiffness measurements assessed by elastography. These results are particularly important given the high prevalence of MASLD in patients with IBD.

Several studies have highlighted that patients with IBD are at a high risk of developing MASLD [6,7,8,9]. However, to our knowledge, only one study has specifically assessed the utility of the FIB-4 index in IBD patients. In a cohort of 321 IBD patients followed over a median of 3.2 years, 108 (34%) developed MASLD, yielding an incidence rate of 9.1 per 100 person-years [10]. In that study, advanced liver fibrosis was identified using a FIB-4 score of ≥2.67. Notably, MASLD development was associated with factors such as disease activity, disease duration, and prior surgery for IBD. Besides, in a previous study on 465 patients with IBD, ultrasonographic presence and degree of steatosis were assessed [27]. Consistent with our findings, there was no significant association between MASLD and IBD behavior, extent, activity, or medication. However, transient elastography was not used in these studies, and the relationship between the FIB-4 index and disease characteristics or liver fibrosis measured by elastography was not investigated.

The most widely accepted theory points to insulin resistance as the primary mechanism driving liver steatosis, and potentially progressing to steatohepatitis, in MASLD [28]. As hepatic steatosis progresses, cellular injury and impaired insulin signaling within the liver worsen inflammatory processes and promote fibrogenesis [29]. Despite this, our study did not find any associations between lipid profiles, insulin resistance, and the FIB-4 index. It is noteworthy that this analysis was conducted in our study exclusively in non-diabetic subjects.

The impact of IBD on insulin sensitivity is still a subject of ongoing discussion in the medical community. Certain research findings suggest that individuals with IBD who are in a state of clinical remission exhibit glucose regulation and insulin response patterns comparable to those observed in healthy subjects. In contrast, other research indicates that the chronic inflammatory state associated with IBD may contribute to the development of insulin resistance in these patients [30].

We do not have a definitive explanation for the lack of association between the FIB-4 index and insulin resistance indices or lipid profiles in our study. One possible reason could be the generally low FIB-4 and elastography values observed in our subjects. It is conceivable that such relationships might become more apparent in advanced stages of liver disease. Additionally, the mechanisms contributing to MASLD in individuals with IBD may differ from those in the general population, meaning insulin resistance might not be the primary factor driving liver disease in this specific group.

In our study, we found an association between the FIB-4 index and the presence of subclinical carotid atherosclerosis. However, this association lost statistical significance when adjusted for traditional cardiovascular risk factors. Additionally, FIB-4 showed a significant positive correlation with the SCORE2 risk assessment calculator. This is supported by the fact that although the independent association between MASLD and cardiovascular disease remains uncertain, some studies have suggested that MASLD is associated with atherosclerotic heart disease, arrhythmias, and heart failure [31].

The correlation between the FIB-4 index and elastography measurements has been previously documented in various studies. In a cohort of 502 subjects with chronic hepatitis C, a strong correlation was observed between these two methods [32]. Similarly, a study involving 122 patients with MASLD reported a comparable correlation coefficient of r = 0.5 (*p* < 0.001) [33]. Furthermore, in a cohort of 418 patients with chronic liver disease of various etiologies, the correlation between FIB-4 and elastography yielded a coefficient of r = 0.505 (*p* < 0.001) [34]. The authors also noted that combining FIB-4 and elastography increased the sensitivity for predicting advanced fibrosis in patients with liver stiffness measurements exceeding 7 kPa. However, this enhanced predictive value was less pronounced when patients with clinically evident cirrhosis were excluded from the analysis [34]. Despite the findings in the aforementioned studies, this correlation was not observed in patients with IBD, where no significant association was found between the FIB-4 index and elastography values. We hypothesize that liver disease in IBD may involve distinct pathophysiological mechanisms compared to other conditions, which could account for the lack of concordance between these two parameters.

While the FIB-4 index was originally developed to predict the onset of hepatic fibrosis, it is frequently used in clinical practice to guide therapeutic interventions aimed at preventing progression to cirrhosis. Based on our findings, however, the FIB-4 index may lack the discriminatory power needed to effectively identify IBD patients who would benefit from such interventions. Chronic inflammation associated with IBD might affect liver enzyme levels and platelet counts in ways that do not directly reflect liver stiffness. For example, inflammation might elevate AST and ALT without proportionally increasing liver fibrosis, influencing the FIB-4 score independently of elastography results.

We recognize that the lack of liver biopsy data could be a limitation of our study. However, large-scale studies involving liver biopsies in patients with IBD are unlikely due to the procedure’s invasive nature, high costs, and the lack of a clear clinical indication for such an invasive approach in this population. Another potential limitation of our study could be the low prevalence of advanced liver disease within our patient cohort. For this reason, we cannot dismiss that the true prevalence may have been underestimated. However, the sample size of our cohort is substantial, and we conducted a thorough characterization of the patients, including individuals with diverse characteristics.

## 5. Conclusions

Our findings indicate that the FIB-4 index does not correlate with disease characteristics or transient elastography values in patients with IBD. This suggests that FIB-4 may not be a reliable predictor of hepatic fibrosis in this population, despite the high prevalence of MASLD among IBD patients. These results highlight the need for further research to identify more effective non-invasive markers for assessing liver fibrosis in IBD patients, especially in the context of MASLD. They also emphasize the importance of being cautious when applying liver fibrosis indexes designed for the general population to specific patient groups, such as those with IBD.

## Figures and Tables

**Table 1:** Characteristics of patients with inflammatory bowel disease.

	IBD Patients
	(*n* = 197)
FIB-4, units	0.93 ± 0.42
Low risk	175 (89)
Indeterminate	22 (11)
High	0 (0)
Male, *n* (%)	107 (54)
Age, years	49 ± 10
Body mass index, kg/m^2^	27 ± 5
Waist-to-hip ratio	0.93 ± 0.07
Hip circumference, cm	101 ± 11
Waist circumference, cm	94 ± 12
Cardiovascular co-morbidity	
Tabacco, *n* (%)	39 (20)
Hypertension, *n* (%)	35 (18)
Diabetes, *n* (%)	11 (6)
Obesity, *n* (%)	55 (28)
Dyslipidemia, *n* (%)	155 (79)
Metabolic syndrome, *n* (%)	69 (35)
Aspirin, *n* (%)	7 (4)
Statins, *n* (%)	21 (11)
IBD related data	
Ulcerative colitis, *n* (%)	67 (34)
Crohn’s disease, *n* (%)	130 (66)
CRP, mg/L	1.8 (0.9–3.8)
Disease duration since diagnosis, years	12 (8–19)
Crohn’s disease related data, *n* (%)	
L1 ileal	56 (43)
L2 colonic	23 (18)
L3 ileocolonic	51 (39)
L4 isolated upper disease	11 (8)
A1 below 16 years	19 (15)
A2 between 17 and 40 years	81 (62)
A3 above 40 years	27 (21)
B1 non-stricturing, non-penetrating	73 (56)
B2 stricturing	46 (35)
B3 penetrating	14 (11)
Harvey–Bradshaw Index	2 (0–4)
Clinical remission	106 (82)
Mildly active disease	14 (11)
Moderately active disease	8 (6)
Severely active disease	1 (1)
CDAI score	39 (7–80)
Asymptomatic remission	116 (89)
Mildly to moderately active Crohn’s disease	10 (8)
Moderately to severely active Crohn’s disease	3 (2)
Severely active to fulminant disease	0 (0)
Ulcerative colitis related data, *n* (%)	
Proctosigmoiditis	7 (10)
Left-sided colitis	23 (35)
Pancolitis	34 (52)
Fecal calprotectin, mcg/g	113 (30–251)
>150	96 (49)
≥150	71 (36)
Partial Mayo score	1 (0–1)
<2	52 (78)
≥2	15 (21)
Perianal disease, *n* (%)	23 (12)
Previous surgery, *n* (%)	55 (28)
Anti-TNF therapy, *n* (%)	58 (29)
Vedolizumab, *n* (%)	5 (3)
Ustekinumab, *n* (%)	8 (4)
Current prednisone, *n* (%)	6 (2)
Prednisone, mg/day	8 (5–20)
Oral mesalazine, *n* (%)	175 (89)
Methotrexate, *n* (%)	22 (11)
Azathioprine, *n* (%)	61 (31)
Tofacitinib, *n* (%)	4 (2)

Data represent mean ± SD or median (interquartile range). CDAI: Crohn’s Disease Activity Index; BMI: body mass index; CRP: C-reactive protein; TNF: tumor necrosis factor; IBD: inflammatory bowel disease; FIB-4: Fibrosis-4 index.

**Table 2:** Inflammatory bowel disease characteristics association with FIB-4 values.

	FIB-4 Index, Units			
	Beta Coefficient (95% Confidence Interval), *p*			
	Univariable		Multivariable	
Age, years	**14 (11–17)**	**<0.001**		
Male	0.09 (−0.02–0.2)	0.11		
Body mass index, kg/m^2^	−0.001 (−0.01–0.01)	0.87		
Hip circumference, cm	−0.003 (−0.008–0.002)	0.26		
Waist circumference, cm	0.002 (−0.003–0.007)	0.39		
Waist-to-hip ratio	**1 (0.4–2)**	**0.003**		
Cardiovascular co-morbidity				
Smoking	0.04 (−0.1–0.2)	0.55		
Hypertension	**0.2 (−0.06–0.4)**	**0.006**		
Diabetes	**0.3 (0.09–0.6)**	**0.007**		
Obesity	0.02 (−0.1–0.2)	0.72		
Dyslipidemia	0.06 (−0.08–0.2)	0.40		
Metabolic syndrome	**0.2 (0.05–0.3)**	**0.007**		
Aspirin	0.2 (−0.1–0.5)	0.24		
Statins	**0.3 (0.1–0.5)**	**0.003**		
IBD related data				
Ulcerative colitis	0.03 (-0.1–0.1)	0.68		
Crohn’s disease	ref.			
CRP, mg/L	**−0.02 (−0.03–(−0.003))**	**0.018**	−0.01 (−0.02–0.0002)	0.055
Duration of disease, years	0.002 (−0.005–0.008)	0.63		
Crohn’s disease related data				
A1 below 16 years	**−0.2 (−0.4–(−0.006))**	**0.043**	−0.05 (−0.2–0.1)	0.54
A2 between 17 and 40 years	−0.05 (−0.2–0.09)	0.48		
A3 above 40 years	**0.2 (−0.08–0.4)**	**0.004**	0.03 (−0.1–0.2)	0.66
L1 ileal	0.03 (−0.1–0.2)	0.69		
L2 colonic	0.7 (−0.1–0.2)	0.48		
L3 ileocolonic	−0.06 (−0.2–0.08)	0.44		
L4 isolated upper disease	−0.1 (−0.4–0.1)	0.31		
B1 non-stricturing, non-penetrating	−0.09 (−0.2–0.05)	0.22		
B2 stricturing	0.1 (−0.04–0.2)	0.15	0.09 (−0.02–0.2)	0.12
B3 penetrating	−0.02 (−0.2–0.2)	0.85		
Harvey–Bradshaw Index	−0.01 (−0.03–0.01)	0.32		
Clinical remission	ref.			
Mildly active disease	0.08 (−0.3–0.1)	0.51		
Moderately active disease	0.03 (−0.3–0.3)	0.85		
Severely active disease	−0.4 (−1–0.4)	0.37		
CDAI score	−0.0006 (−0.002–0.0003)	0.21		
Asymptomatic remission	ref.			
Mildly to moderately Crohn’s disease	−0.1 (−0.4–0.1)	0.35		
Moderately to severely Crohn’s disease	−0.1 (−0.6–0.3)	0.61		
Severely active to fulminant disease	ref.			
Ulcerative colitis related data, *n* (%)				
Proctosigmoiditis	−0.3 (−0.6–0.1)	0.16	−0.2 (−0.5–0.2)	0.33
Left-sided colitis	0.1 (−0.1–0.3)	0.41		
Pancolitis	−0.007 (−0.2–0.2)	0.95		
Score partial Mayo	0.008 (−0.07–0.08)	0.84		
<2	ref.			
≥2	0.2 (−0.08–0.04)	0.17	0.1 (−0.1–0.3)	0.42
Fecal calprotectin, mcg/g	-0.0007 (−0.0002–0.00007)	0.30		
>150	ref.			
≥150	−0.03 (−0.1–0.09)	0.66		
Perianal disease, *n* (%)	−0.02 (−0.2–0.2)	0.86		
Previous surgery, *n* (%)	−0.09 −0.2–0.04)	0.16	−0.05 (−0.2–0.06)	0.41
Current prednisone, *n* (%)	−0.06 (−0.4–0.3)	0.74		
Prednisone, mg/day	−0.009 (−0.06–0.04)	0.55		
Oral mesalazine, *n* (%)	0.05 (−0.07–0.2)	0.39		
Methotrexate, *n* (%)	−0.0003 (−0.2–0.2)	0.99		
Azathioprine, *n* (%)	−0.04 (−0.2–0.09)	0.58		
Anti-TNF therapy, *n* (%)	−0.02 (−0.2–0.1)	0.71		
Ustekinumab, *n* (%)	−0.3 (−0.6–0.006)	0.055	−0.09 (−0.3–0.2)	0.47
Vedolizumab, *n* (%)	−0.1 (−0.5–0.2)	0.52		
Tofacitinib, *n* (%)	−0.3 (−0.07–0.1)	0.20		

In this analysis, the FIB-4 index is the dependent variable. BMI: body mass index; CRP: C-reactive protein; TNF: tumor necrosis factor; CDAI: Crohn’s Disease Activity Index. IBD: inflammatory bowel disease; FIB-4: Fibrosis-4 index. CDAI was categorized as 0 to 149: asymptomatic remission; 150 to 220 points: mildly to moderately active. Multivariable analysis was adjusted for age, sex, diabetes, hypertension, statin intake, and waist-to-hip ratio. Significant *p* values are depicted in bold.

**Table 3:** Cardiovascular features’ relation to MDA serum levels.

		FIB-4 Index, Units			
		Beta Coefficient (95% Confidence Interval), *p*			
		Univariable		Multivariable	
Carotid intima-media assessment					
Carotid plaque, *n* (%)	68 (35)	**0.2 (0.1–0.4)**	**<0.001**	−0.002 (−0.1–0.1)	0.97
Bilateral plaque, *n* (%)	35 (18)	**0.3 (0.2–0.5)**	**<0.001**	0.07 (−0.08–0.2)	0.36
cIMT, mm	0.644 ± 0.137	**1 (0.7–0.2)**	**<0.001**	0.3 (−0.2–0.7)	0.20
SCORE2 risk, %	2.3 (1.0–4.4)	**0.06 (0.04–0.07)**	**<0.001**		
Low category	140 (71)	ref.			
Moderate category	49 (25)	0.1 (−0.02–0.2)	0.084		
High category	7 (4)	**0.6 (0.3–0.9)**	**<0.001**		
Insulin resistance indices *					
Glucose, mg/dL	95 ± 19	0.001 (−0.002–0.004)	0.47		
C peptide, ng/ml	0.72 ± 0.43	−0.003 (−0.04–0.04)	0.89		
Insulin, µU/mL	6.7 (4.8–11.1)	0.001 (−0.006–0.009)	0.70		
HOMA2-B%	119 ± 49	−0.0003 (−0.001–0.0007)	0.52		
HOMA2-IR	0.86 (0.62–1.46)	0.01 (−0.04–0.07)	0.69		
HOMA2-S%	131 ± 82	−0.00005 (−0.0006–0.0005)	0.86		
Lipid profile					
Cholesterol, mg/dL	201 ± 49	0.0009 (−0.002–0.002)	0.12	0.0002 (−0.0008–0.001)	0.69
LDL, mg/dL	116 ± 40	0.0007 (−0.0008–0.002)	0.35		
HDL, mg/dL	57 ± 18	−0.001 (−0.005–0.002)	0.38		
LDL/HDL ratio	2.18 ± 0.86	0.05 (−0.02–0.01)	0.16	0.02 (−0.04–0.08)	0.48
Non-HDL chol., mg/dL	146 ± 43	**0.001 (0.0001–0.003)**	**0.032**	0.005 (−0.0007–0.002)	0.40
Triglycerides, mg/dL	151 ± 89	**0.001 (0.0004–0.002)**	**0.002**	0.0005 (−0.0001–0.001)	0.10
Apo B/Apo A ratio	0.69 ± 0.22	0.2 (−0.06–0.5)	0.13	0.05 (−0.2–0.3)	0.68
Apolipoprotein A1, mg/dL	162 ± 37	0.005 (−0.001–0.002)	0.53		
Apolipoprotein B, mg/dL	108 ± 32	**0.002 (0.0002–0.004)**	**0.028**	0.0005 (−0.001–0.002)	0.56
Atherogenic index	3.8 ± 1.2	0.05 (0.003–0.1)	0.036	0.02 (−0.02–0.07)	0.29
Lipoprotein (a), mg/dL	26 (8–88)	0.00001 (−0.0007–0.0008)	0.96		
Apolipoprotein C-III, mg/dL	3.5 (2.8–4.4)	0.01 (−0.02–0.04)	0.40		

In this analysis, the FIB-4 index is the dependent variable. FIB-4: Fibrosis-4 index; cIMT: carotid intima-media thickness; LDL: low-density lipoprotein; HDL: high-density lipoprotein; SCORE2: Systematic Coronary Risk Evaluation-2; HOMA2: homeostatic model assessment. Multivariable analysis was adjusted for age, sex, diabetes, hypertension, statin intake, and waist-to-hip ratio. * The relationship between insulin resistance indices and FIB4 was only performed in non-diabetic patients with a glucose <110 (*n* = 155). Significant *p* values are depicted in bold.

**Table 4:** Relationship between FIB-4, liver elastography, and liver ultrasound.

		FIB-4, Units		Elastography, kPa	
		Beta Coefficient (95% CI), *p*			
FIB-4, units	0.93 ± 0.42			0.2 (−0.9–1)	0.70
Low risk	175 (89)			ref.	
Indeterminate	22 (11)			0.4 (−1–2)	0.55
High	0 (0)				
Elastography, kPa	5.27 ± 2.89	0.004 (−0.02–0.03)	0.70		
F0-F1	140 (86)	ref			
F2	14 (9)	0.04 (−0.2–0.3)	0.73		
F3	5 (3)	0.2 (−0.2–0.5)	0.35		
F4	3 (2)	−0.1 (−0.6–0.3)	0.59		
Ultrasound grade of steatosis					
Absence	89 (51)	ref.		ref.	
Mild	53 (31)	0.1 (−0.02–0.3)	0.10	0.8 (−0.2–2)	0.11
Moderate	27 (16)	0.1 (−0.06–0.3)	0.20	**3 (2–5)**	**<0.001**
Severe	4 (2)	0.2 (−0.3–0.6)	0.47	4 (−1–9)	0.13

In this analysis, the FIB-4 index and elastography are both independent and dependent variables when applied. For the analysis of ultrasound grades of steatosis, these are independent variables. FIB-4: Fibrosis-4 Index. Significant *p* values are shown in bold.

## Data Availability

The original contributions presented in the study are included in the article, and further inquiries can be directed to the corresponding authors.
